# Treatment challenges in patients with early acute massive pulmonary thrombosis embolism (PTE) after lung cancer surgery: A case report

**DOI:** 10.1097/MD.0000000000025371

**Published:** 2021-04-09

**Authors:** Jian Shen, Mi Zhou, Weihua Shao, Haiyun Dai

**Affiliations:** a Department of Cardiology; b Department of Respiratory and Critical Care Medicine, The First Affiliated Hospital of Chongqing Medical University, Chongqing, China.

**Keywords:** case report, early acute massive pulmonary thrombosis embolism, hemorrhage, post lung cancer surgery, thrombolysis

## Abstract

**Introduction:**

Early acute massive pulmonary thrombosis embolism (PTE) after lung cancer surgery is one of the most fatal surgical complications. It is often accompanied by shock and hypotension, with high mortality rate. Due to surgical wounds, patients with early acute massive PTE after lung cancer surgery have a high risk of thrombolytic bleeding, which renders treatment more challenging and there is currently no standard protocol on how to safely and effectively treat these patients in the clinic.

**Patient concerns:**

A 66-year-old woman after video-assisted thoracoscopic surgery for lung cancer, experienced sudden severe dyspnea, shock and hypotension with high D-Dimer, changed electrocardiogram (ECG), right ventricular dilatation, severe tricuspid regurgitation, and raised pulmonary arterial pressure on ultrasonic cardiogram (UCG), thromboses found on Ultrasonography of lower extremity vein.

**Diagnosis:**

Because of her clinical manifestations and results of bedside auxiliary examinations, the patient was finally diagnosed with acute high-risk PTE after lung cancer surgery.

**Interventions:**

1.5 hours after onset of symptoms, thrombolysis using a continuous micropump infusion of 20,000 units/kg urokinase into the peripheral vein for 2 hours was initiated for this patient.

**Outcomes:**

The patient died of massive hemorrhage after thrombolysis.

**Lessons:**

Treatment for patients with early acute PTE after lung cancer surgery is challenging due to a high risk of thrombolytic bleeding at the surgical site. Real-time monitoring of vital signs during thrombolysis and catheter-directed thrombolysis are recommended for these patients, in order to use the minimum drug dosage for quick curative effects and a low risk of bleeding.

## 1 Introduction

With advances in understanding of the etiology of pulmonary thrombosis embolism (PTE) as well as improvements of diagnostic methods, the reported incidence rates of PTE after lung cancer surgery have been gradually declining.^[1]^ Currently, the main treatment option for acute massive PTE is thrombolysis. Lung cancer patients who have undergone surgery are particularly susceptible to PTE as it is associated with risk factors such as malignant tumors, long operation times, and damage to the walls of blood vessels during surgery. Sakuragi et al^[2]^ have reported a PTE incidence of 19.5% after single lobectomy and pneumonectomy, with mortality rates of 22% and 50%, respectively. Due to its rapid progression, accompanied by shock and hypotension as well as the high risk of thrombolytic bleeding, the mortality rates reached up to of 44% to 93%^[3]^ for patients with early acute massive PTE after lung cancer surgery, thus the treatment strategy is challenging. Here, we provide a brief review of the literature and report on the case of a patient who died of massive hemorrhage due to thrombolytic therapy for early acute massive PTE after lung cancer surgery. We have assessed the optimal treatment strategies for these patients in order to more effectively treat this fatal complication in the future and to improve the prognosis of patients.

## 2 Case report

**Figure 1:**
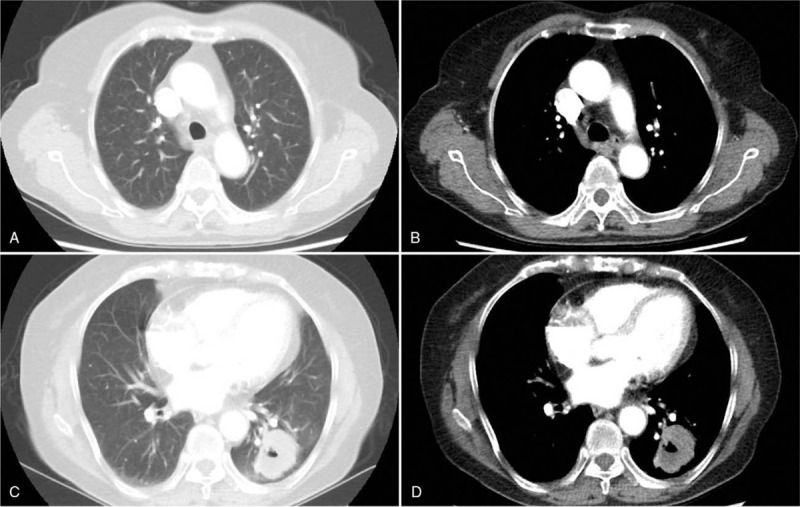
Mediastinal lymph node enlargement in the pulmonary window (A) and mediastinal window(B); a 41 × 38 mm mass shadow with a cavity of the left inferior pulmonary lobe in the pulmonary window (C) and mediastinal window (D) at the enhanced chest-CT.

**Figure 2:**
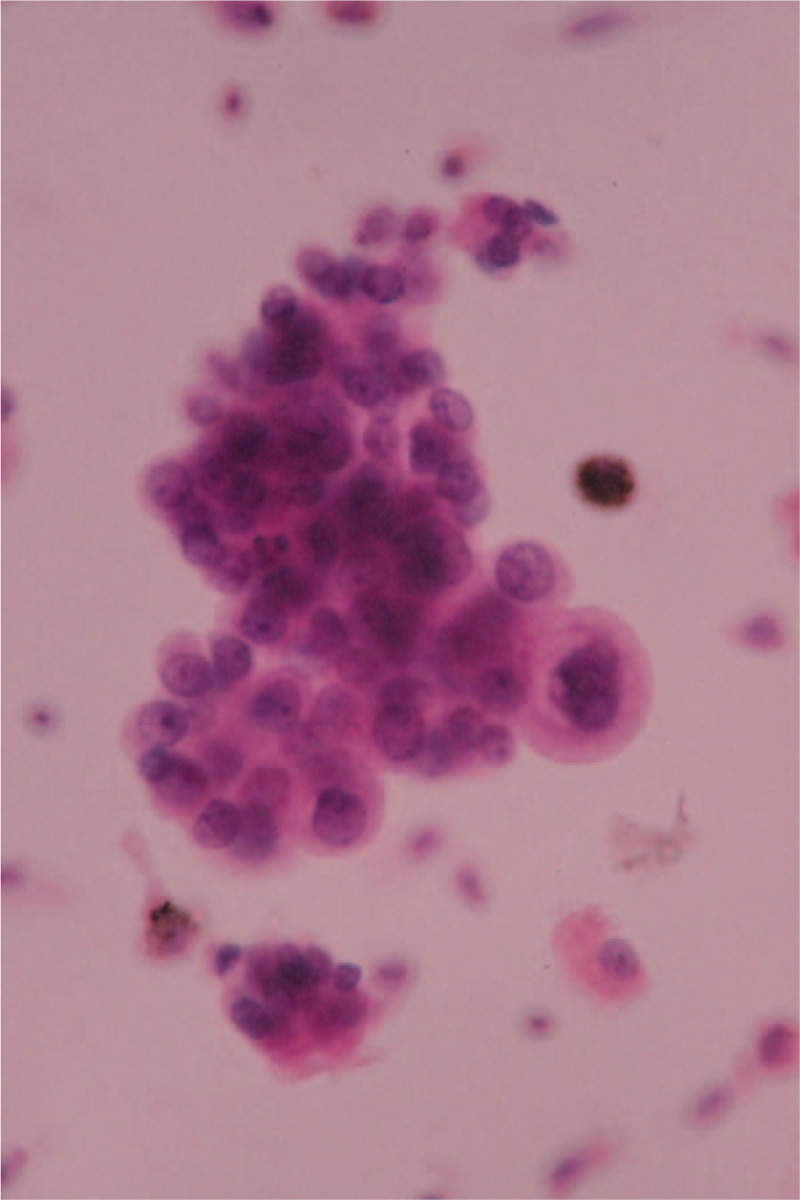
Adenocarcinoma cells in the bronchoscopic aspiration.

**Figure 3:**
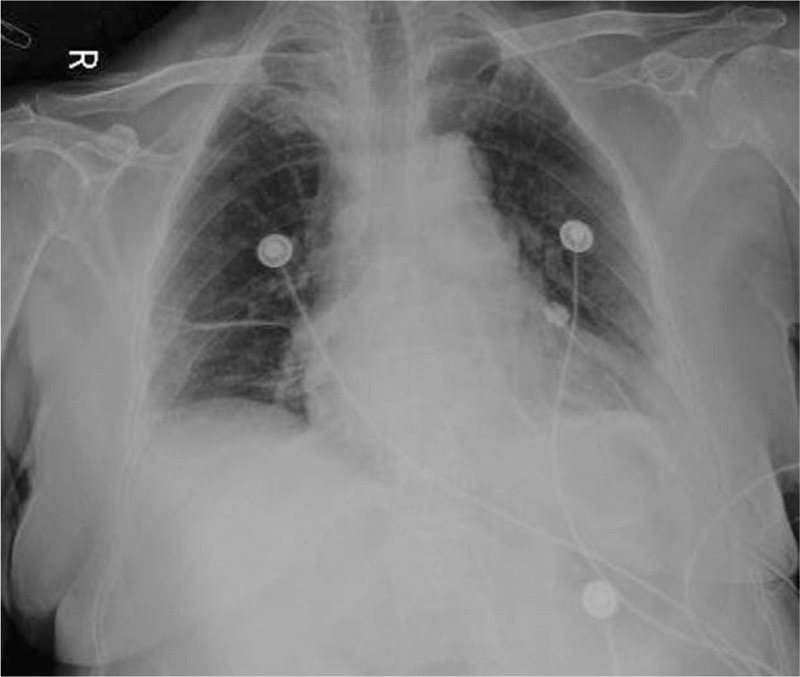
The first postoperative chest X-ray, performed 4 days after the operation.

**Figure 4:**
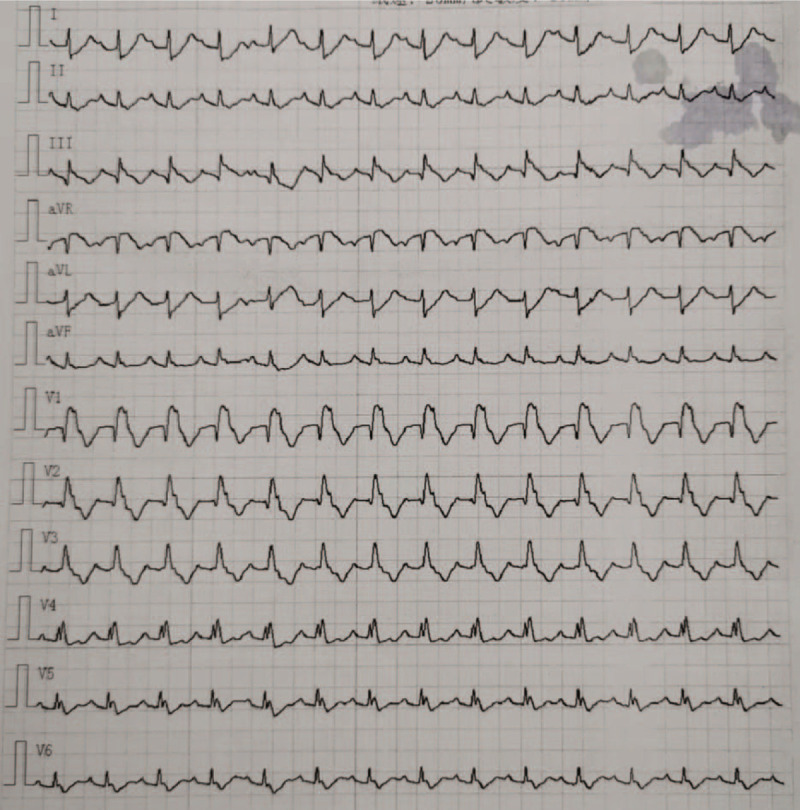
ECG performed during a sudden change of the patient's condition.

**Figure 5:**
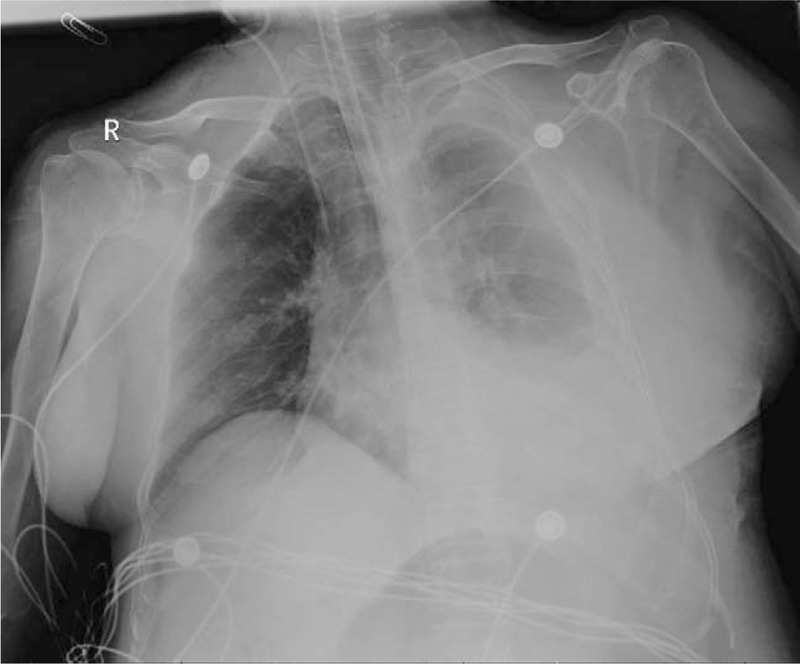
The second chest X-ray displaying a left thoracic hemorrhage.

A 66-year-old asymptomatic Chinese woman weighing 47 kg without any abnormalities in her medical history underwent a chest computed tomography (CT) scan for a routine health checkup, during which a 41 × 38 mm mass shadow of the left inferior pulmonary lobe with mediastinal lymph node enlargement was found (Fig. 1A-D). Further tests revealed adenocarcinoma cells in bronchoscopic aspiration (Fig. 2) and histopathological examinations resulted in diagnosis of invasive adenocarcinoma (T2bN2M0 IIIA). After admission, no obvious abnormalities were found by electrocardiogram (ECG) and ultrasonic cardiogram (UCG) tests, and therefore ultrasonography of the lower limb vessels was not performed. After exclusion of surgical contraindications, video-assisted thoracoscopic surgery for lung cancer was carried out. Following surgery, the patient received standard care including postoperative anti-infection, atomization, and expectoration, and a chest X-ray re-examination was performed 4 days after surgery (Fig. 3). The patient experienced severe dyspnea during the following night and upon physical examination, the following parameters were measured: blood pressure (BP) 80/54 mm Hg, heart rate (HR) 45 to 50 bpm, pulse oxygen saturation (SpO2) 60% to 80% (oxygen flow rate 5 L/minute), moist rales scattered in the right lower lung. Blood-gas analysis revealed a pH of 7.29, PO2 of 43 mm Hg, PCO2 of 50 mm Hg SaO2 of 72%, and 6.2 mmol/L Lac. The patient lost consciousness and the carotid pulse was impalpable, which was treated with immediate chest compression, tracheal intubation and continuous infusion of dopamine and norepinephrine. Due to hemodynamic instability, the patient did not receive computed tomographic pulmonary angiography. The patient's D-Dimer was 26.14 mg/L. ECG tracing are shown in Figure 4. UCG revealed a right ventricular dilatation of 24 mm, severe tricuspid regurgitation, and the presence of a mean pulmonary arterial pressure of 63 mm Hg. Ultrasonography of lower extremity vein revealed thromboses of the right superficial femoral vein and bilateral intermuscular veins. The patient was therefore diagnosed with acute high-risk PTE; 1.5 hours after onset of symptoms, thrombolysis using a continuous micropump infusion of 20,000 units/kg urokinase into the peripheral vein for 2 hours was initiated with consent from patient's family. The patient gradually regained consciousness, and a BP of 108/57 mm Hg was measured (most likely due to continuous pumping of pressor drugs). The patient revealed a HR of 120 to 140 bpm and SpO2 had risen to 90% to 100%. Blood-gas analysis revealed a pH of 7.17, PO2 of 370 mm Hg, PCO2 of 49 mm Hg, SaO2 of 100%, and >15 mmol/L Lac (invasive mechanical ventilation resulted in SpO2 99%). Coagulation function tests revealed obvious abnormalities. Six hours after thrombolysis, bleeding in the mouth, skin of the palms, and surgical incision site occurred. Hemoglobin (Hb) decreased significantly from an initial 101 g/L to 65 g/ L. Within the next 2 days, the patient received 2600 ml of erythrocyte transfusion, 2400 ml of plasma transfusion and 18 units of cryofibrinogen. However, the bleeding did not stop. Repeat Hb measurement revealed an Hb concentration of 60 g/L and no improvement in coagulation function. The second postoperative chest X-ray showed left thoracic hemorrhage (Fig. 5), and the patient ultimately died of massive hemorrhage.

## 3 Discussion

As there is currently no completely safe and effective treatment for high-risk PTE, the first choice of treatment remains thrombolytic therapy. However, 20% of PTE have been reported to experience bleeding after intravenous thrombolysis,^[4]^ with the risk of bleeding being higher due to surgical wounds. This was also the case with the patient reported on here, who died of massive hemorrhage following thrombolytic therapy. It has been reported that over 50% of patients receiving thrombolysis develop massive hemorrhage within the first week after surgery.^[5]^ While there are no incidence reports on hemorrhage in patients treated with thrombolysis after lung cancer surgery, the incidence is estimated to be even higher in this patient population. Because postoperative thrombolysis is associated with a high incidence of massive hemorrhage which can be life threatening in clinic, reducing the bleeding complications following thrombolytic therapy is key for the successful treatment of these patients. Without new and revolutionary drugs, the optimization of dosage and administration route of thrombolytic drugs might reduce the bleeding complications.

It has been reported that the risk of bleeding may be increased in patients with moderate- to high-risk acute PTE treated with conventional thrombolytic drugs. However, reducing the dose of thrombolytic drugs, including rt-PA, can significantly reduce the risk of bleeding without increasing the risk for PTE recurrence or mortality.^[6,7]^ Therefore, it has been suggested that a continuous intravenous drip of thrombolysis at half the normal dose (50 mg rt-PA, 20,000 units/kg urokinase, recombinant streptokinase 1.5 million units) for 2 hours could be recommended for thrombolytic therapy in patients with acute PTE. Vascular intervention techniques may not be as sophisticated in nonspecialist hospitals and therefore peripheral intravenous thrombolysis is an indispensable and relatively easily administered treatment. Patients with early acute massive PTE following lung cancer surgery have high risk of bleeding due to surgical wounds. In order to reduce the risk of bleeding, monitoring the HR, BP, and SpO2 in real time during thrombolysis is recommended. Once shock, hypotension, and hypoxemia are corrected, administration of thrombolytic drugs should be stopped, and only the minimum dose of thrombolytic drugs required to achieve thrombolysis should be applied to prevent massive bleeding. However, more clinical research is needed to investigate treatment of PTE in these high-risk patients further.

Catheter-directed thrombolysis (CDT) can help to reduce the required dosage of thrombolytic drugs and hemorrhage. A retrospective study conducted by Bloomer et al. found that CDT is safe for high-risk PTE patients.^[8]^ Regardless of thrombolytic route, bleeding at surgical sites is the most critical issue to consider when treating PTE with thrombolysis. At present, there is no large-scale high-level clinical study assessing the optimal thrombolytic route for patient with PTE after lung cancer surgery. The choice of the appropriate thrombolytic route depends on the specific situation of the patient and the medical equipment and techniques of the hospital. A meta-analysis of 35 nonrandomized controlled trials showed that the success rate of CDT is over 85%, with a low incidence of complications,^[9]^ and several other clinical studies have likewise suggested a high success rate for CDT, which could be used as an effective alternative to intravenous thrombolysis.^[10–12]^ Based on the literature above, it may be suggested that CDT should be considered for patients developing acute high-risk PTE within 1 week of lung cancer surgery instead of intravenous thrombolysis, due to the high risk of fatal bleeding. CDT can also be assisted by catheter-mediated thrombectomy, which includes percutaneous mechanical crushing and thrombectomy, negative pressure aspirated thrombectomy, rheologic thrombectomy, and catheter mechanical thrombectomy.^[13]^ Therefore, an emergency multidisciplinary-team (MDT) discussion involving personnel from thoracic surgery, vascular surgery, respiratory departments, and intensive care unit, should have been considered for the patient reported on here who developed early acute massive PTE within 1 week after lung cancer surgery. Achieving multidisciplinary cooperation in order to get the opportunity to perform CDT may have changed the outcome of this patient, avoiding massive hemorrhage and death. As for intravenous thrombolysis, in order to use the minimum dose of thrombolytic drugs achieving thrombolysis but preventing massive bleeding, monitoring of the HR, BP, and SpO2 in real time, as well as observing pulmonary perfusion by intermittent angiography during CDT should be performed. Once vital signs improve, the main pulmonary blood flow recovers and pulmonary artery pressure decreases, CDT should be discontinued, with bridging heparin anticoagulant therapy.^[14]^

Apart from thrombolysis, surgical methods, such as embolectomy, may be used to treat PTE.^[15]^ However, also these techniques face high mortality rates. It has been reported that extracorporeal membrane oxygenation (ECMO) can provide the opportunity to perform CDT or surgical thrombectomy in patients with unstable vital signs, and may also be used as bridging therapy after thrombolysis and CDT.^[16]^ If there is a stump or breakage of pulmonary vessels that is not mechanically closed during lung cancer surgery, the surgeon is required to consider this before CDT and monitor active bleeding at the surgical site during CDT, taking appropriate measures to prevent complications. Anticoagulant therapy should be given after the initial treatment, which is also suggested to be given to patients with deep vein thrombosis risk factors life-long.

**Figure 6:**
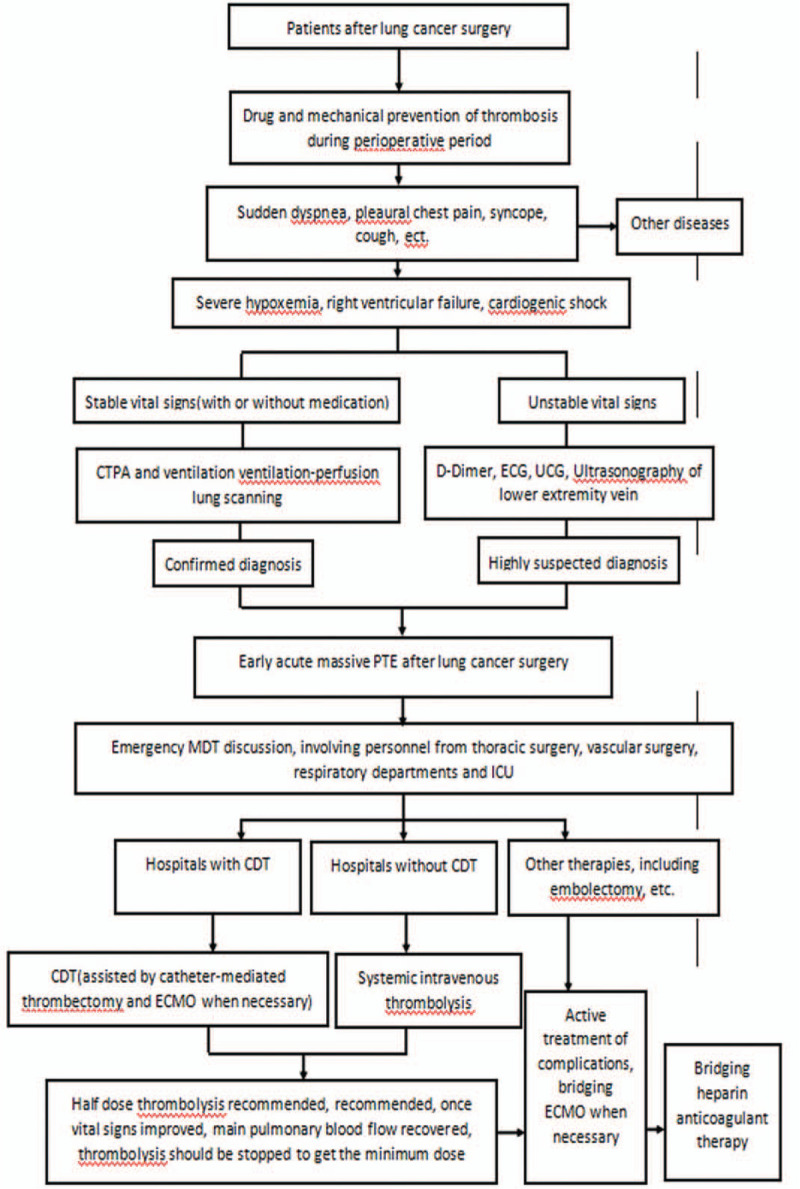
Diagnosis and treatment process for patients with early acute massive PTE after lung cancer surgery.

Apart from the treatment strategies mentioned above, a multidisciplinary team is suggested to be set up with standardized diagnosis and treatment process (Fig. 6) in qualified hospitals in order to achieve early prevention, diagnosis, and treatment for patient with early acute massive PTE after lung cancer surgery.

## 4 Conclusion

Treatment for patients with early acute PTE after lung cancer surgery is challenging due to a high risk of thrombolytic bleeding at the surgical site. Once diagnosis of early acute massive PTE after lung cancer surgery is confirmed, a MDT discussion should be held as soon as possible, and the rapid, appropriate choice of treatment may be lifesaving. Through real-time monitoring of vital signs during thrombolysis, thrombolysis can be stopped immediately when vital signs have improved or stabilized in order to use the minimum dose of the thrombolytic drug required to achieve an effective therapeutic effect. CDT is recommended for treatment of early acute massive PTE after lung cancer surgery over intravenous thrombolysis, with the advantages of accurate administration, easy control of the drug dosage, quick curative effects and a low risk of bleeding. However, more research will be needed in the future to address the treatment challenges for PTE in these patients.

## Acknowledgments

The authors would like to thank Professor Hong Chen for critically reviewing the manuscript.

## Author contributions

**Conceptualization:** Haiyun Dai.

**Data curation:** Weihua Shao.

**Funding acquisition:** Haiyun Dai.

**Investigation:** Mi Zhou.

**Supervision:** Haiyun Dai.

**Writing – original draft:** Jian Shen.

**Writing – review & editing:** Haiyun Dai.

## References

[1] Shonyela FS Yang S Liu B. Postoperative acute pulmonary embolism following pulmonary resections. Ann Thorac Cardiovasc Surg 2015;21:409–17. 26354232 10.5761/atcs.ra.15-00157 PMC4904848

[2] Sakuragi T Sakao Y Furukawa K. Successful management of acute pulmonary embolism after surgery for lung cancer. Eur J Cardiothorac Surg 2003;24:580–7. 14500078 10.1016/s1010-7940(03)00392-0

[3] Kilic D Akin S Findikcioglu A. Low-molecular-weight heparin for treatment of submassive pulmonary embolism after pneumonectomy. Gen Thorac Cardiovasc Surg 2007;55:287–9. 17679257 10.1007/s11748-007-0124-8

[4] Schmid C Zietlow S Wagner TO. Fulminant pulmonary embolism: symptoms, diagnostics, operative technique, and results. Ann Thorac Surg 1991;52:1102–5. discussion 1105-7. 1953130 10.1016/0003-4975(91)91288-7

[5] Condliffe R Elliot CA Hughes RJ. Management dilemmas in acute pulmonary embolism. Thorax 2014;69:174–80. 24343784 10.1136/thoraxjnl-2013-204667 PMC3913120

[6] Wang C Zhai Z Yang Y. Efficacy and safety of low dose recombinant tissue-type plasminogen activator for the treatment of acute pulmonary thromboembolism: a randomized, multicenter, controlled trial. Chest 2010;137:254–62. 19741062 10.1378/chest.09-0765 PMC7126994

[7] Zhang Z Zhai ZG Liang LR. Lower dosage of recombinant tissue-type plasminogen activator (rt-PA) in the treatment of acute pulmonary embolism: a systematic review and meta-analysis. Thromb Res 2014;133:357–63. 24412030 10.1016/j.thromres.2013.12.026

[8] Bloomer TL El-Hayek GE McDaniel MC. Safety of catheter-directed thrombolysis for massive and submassive pulmonary embolism: results of a multicenter registry and meta-analysis. Catheter Cardiovasc Interv 2017;89:754–60. 28145042 10.1002/ccd.26900

[9] Kuo WT Gould MK Louie JD. Catheter-directed therapy for the treatment of massive pulmonary embolism: systematic review and meta-analysis of modern techniques. J Vasc Interv Radiol 2009;20:1431–40. 19875060 10.1016/j.jvir.2009.08.002

[10] Kuo WT Banerjee A Kim PS. Pulmonary embolism response to fragmentation, embolectomy, and catheter thrombolysis (PERFECT): initial results from a prospective multicenter registry. Chest 2015;148:667–73. 25856269 10.1378/chest.15-0119

[11] Aklog L Williams CS Byrne JG. Acute pulmonary embolectomy: a contemporary approach. Circulation 2002;105:1416–9. 11914247 10.1161/01.cir.0000012526.21603.25

[12] Bajaj NS Kalra R Arora P. Catheter-directed treatment for acute pulmonary embolism: systematic review and single-arm meta-analyses. Int J Cardiol 2016;225:128–39. 27718446 10.1016/j.ijcard.2016.09.036

[13] Taslakian B Sista AK. Catheter-directed therapy for pulmonary embolism: patient selection and technical considerations. Interv Cardiol Clin 2018;7:81–90. 29157527 10.1016/j.iccl.2017.08.002

[14] Jing S Zhou J Lu Q. Experience of interventional thrombolysis therapy for massive pulmonary thrombosis embolism after video-assisted thoracoscopic surgery for lung cancer. Zhongguo Fei Ai Za Zhi 2018;21:779–83. 30309430 10.3779/j.issn.1009-3419.2018.10.08 PMC6189031

[15] Lehnert P Møller CH Mortensen J. Surgical embolectomy compared to thrombolysis in acute pulmonary embolism: morbidity and mortality. Eur J Cardiothorac Surg 2017;51:354–61. 28186234 10.1093/ejcts/ezw297

[16] Carroll BJ Shah RV Murthy V. Clinical Features and outcomes in adults with cardiogenic shock supported by extracorporeal membrane oxygenation. Am J Cardiol 2015;116:1624–30. 26443560 10.1016/j.amjcard.2015.08.030

